# How case representations of medical students change during case processing – Results of a qualitative study

**DOI:** 10.3205/zma001187

**Published:** 2018-08-15

**Authors:** Leah Theresa Braun, Benedikt Lenzer, Jan Kiesewetter, Martin R. Fischer, Ralf Schmidmaier

**Affiliations:** 1 Ludwig-Maximilians-University (LMU), Klinikum der Universität München, Medizinische Klinik und Poliklinik IV, Munich, Germany; 2 Klinikum der LMU München, Institut für Didaktik und Ausbildungsforschung in der Medizin, Munich, Germany

**Keywords:** diagnostic competence, clinical reasoning, undergraduate medical education

## Abstract

**Objective: **Representations are mental summaries of a clinical case and help in understanding a clinical problem. However, it is still largely unknown which clinical information medical students include in their case representations. In this study, therefore, the structure and quality of students’ case representations were examined to better understand the diagnostic process and its relationship to diagnostic accuracy. What information do medical students include in their representations and is there an association between this information and the diagnostic accuracy?

**Method:** 43 medical students in the fourth and fifth clinical year worked on four clinical cases. During the diagnostic process, they were asked three times per case to write a case representation. 516 representations were qualitatively evaluated using a content-based coding scheme. An analysis was made of the nature and composition of the clinical information. In addition, the association between the general representation structure and the correct case solution was examined.

**Results: **At the beginning, students include most of the clinical information in their representation (66%), but as the case progresses, they begin to select the information offered (2nd representation 42%, 3rd representation 38%). The length of the representation (number of words) does not correlate with the correct case solution (r=-0.08-0.31). The representations do not depend on the case difficulty but have a significant individual component: the representations written by a student are formally very similar in all four cases

(r=0.60-0.86).

**Conclusion:** Medical students can select the relevant clinical information and include it in their case representations. Lack of representation does not seem to be a reason for misdiagnosis; Students’ deficits in diagnosis are more likely due to knowledge gaps.

## Zusammenfassung

**Zielsetzung: **Repräsentationen sind mentale Zusammenfassungen eines klinischen Falls und helfen beim Verständnis eines klinischen Problems. Es ist bisher allerdings noch weitgehend unbekannt, welche klinischen Informationen Medizinstudierende in ihre Fallrepräsentationen einschließen. In dieser Studie wurden deshalb die Struktur und die Qualität von studentischen Fallrepräsentationen untersucht, um den Diagnoseprozess und seine Beziehung zur Diagnoserichtigkeit besser zu verstehen: Welche Informationen schließen Medizinstudierende in ihre Repräsentationen ein und besteht eine Assoziation zwischen diesen Informationen und der Diagnoserichtigkeit?

**Methodik: **43 Medizinstudierende im vierten und fünften klinischen Jahr bearbeiteten vier klinische Fälle. Während des Diagnoseprozesses wurden sie dreimal pro Fall aufgefordert, eine Fallrepräsentation zu schreiben. 516 Repräsentationen wurden mit Hilfe eines inhalts-basierten Codierschemas qualitativ ausgewertet. Es erfolgte eine Analyse der Art und der Zusammensetzung der klinischen Informationen. Außerdem wurde die Assoziation zwischen der allgemeinen Repräsentationsstruktur und der korrekten Falllösung überprüft.

**Ergebnisse: **Studierende inkludieren zu Beginn die meisten klinischen Informationen in ihre Repräsentation (66%), aber im Fallverlauf beginnen sie, die angebotenen Informationen zu selektieren (2. Repräsentation 42%, 3. Repräsentation 38%). Die Länge der Repräsentation (Anzahl der Wörter) korreliert nicht mit der korrekten Falllösung (r=-0.08–0.31). Die Repräsentationen hängen nicht von der Fallschwierigkeit ab, sondern haben eine signifikante individuelle Komponente: Die Repräsentationen, die von einem Studierenden geschrieben wurden, ähneln sich in allen vier Fällen formal sehr stark (r=0.60–0.86).

**Schlussfolgerung: **Medizinstudierende können die relevanten klinischen Informationen selektieren und in ihre Fallrepräsentationen einschließen. Mangelnde Repräsentationsfähigkeit scheint kein Grund für Fehldiagnosen zu sein; Studentische Defizite beim Diagnostizieren sind eher auf Wissenslücken zurückzuführen.

## Introduction

Diagnosis is one of the central tasks in everyday clinical practice. A characteristic of the medical expert is the ability to diagnose correctly. The development of expertise has been studied for many years [1], [2]: the illness-script theory is a widely accepted model for explaining the diagnostic behavior of experts [2]. This theory states that an expert has a particular concept of any disease that includes the epidemiology, the symptoms, and the enabling factors of a disease [3]. Representation is a relevant aspect of the diagnostic process. It can be defined as a mental summary of the case by the physician/student. The problem of the case is represented in such a way that the cognitive processes can be used to understand and solve it [4]. Therefore, representation is one of the cognitive steps that correlates with a correct case solution [5]. Charlin et al. distinguish an initial representation, a dynamic representation as well as a final representation in the course of the diagnostic process [6]. A good mental representation of the clinical problem is necessary to activate relevant prior-knowledge and to compare different differential diagnoses so that finally the correct diagnosis can be made. The initial representation of the problem is modified and enriched by new information, so that at the end of the diagnostic process the case is comprehensively represented [7]. Physicians use representations in their everyday clinical routine as part of the diagnostic process, but also in other situations, such as the ward round [8] or handovers [9], [10], [11]. Representations as a key element in the diagnostic process [6], [12] allow the physician to compare his illness script with the current appearance of the patient; and the student, to compare the patient case with his theoretical knowledge. It is expected that experts will evaluate whether the initial representation matches one of their illness scripts, which is the starting point for further (selective) data acquisition and dynamic representation in the decision-making process.

The structural aspects of representations have already been analyzed; semantic and symptom-oriented processes can be distinguished [13]. Representation begins with semantic transformation [14]. The clinical data are transformed with the help of so-called semantic qualifiers, which represent an abstraction of the clinical findings (e.g. last night → acute onset) and contribute to the diagnostic accuracy [15], [16]. It has already been shown that student representations can be improved by encouraging the use of semantic transformations [12]. Therefore, it seems promising to use representations to improve the diagnostic competence of medical students. In a recent study, we found that a prompt to write representations improves, for example, the diagnostic efficiency of students, but not the diagnostic accuracy [17].

So far, there is no empirical data on the information categories of representations and the development of them during the student diagnostic process. It is unclear which concrete information, such as which anamnestic data or results of various examinations, are contained in representations. Moreover, there is no data on how comprehensive the representations are, that is, whether all given information is included or, in fact, merely a selection of the data is in the representations.

However, a better understanding is indispensable as a basis for interventions, such as scaffolding in the form of structured reflection or representation. This knowledge can help to control prompts for representations or case summaries and adapt them better to student deficits.

These considerations lead to the following research questions:

- Research question 1: How much and what information is included in repeated case representations in simple, intermediate and difficult clinical cases and how does it develop during the diagnostic process?
- Research question 2: Is there an association between information categories and the structure of representations and the correct diagnosis?

## Method

### Study design, participants and procedure

This article describes the results of a qualitative analysis of an intervention study that examined the influence of representations on the diagnostic competence of medical students and the underlying mechanisms [17], [18]. In the study, a control group and an intervention group, which received representation prompts, were compared in terms of diagnostic accuracy and diagnostic efficiency. In addition, the diagnostic errors of the students were qualitatively evaluated. This article is a secondary partial analysis of the intervention arm of this study.

**Figure 1:**
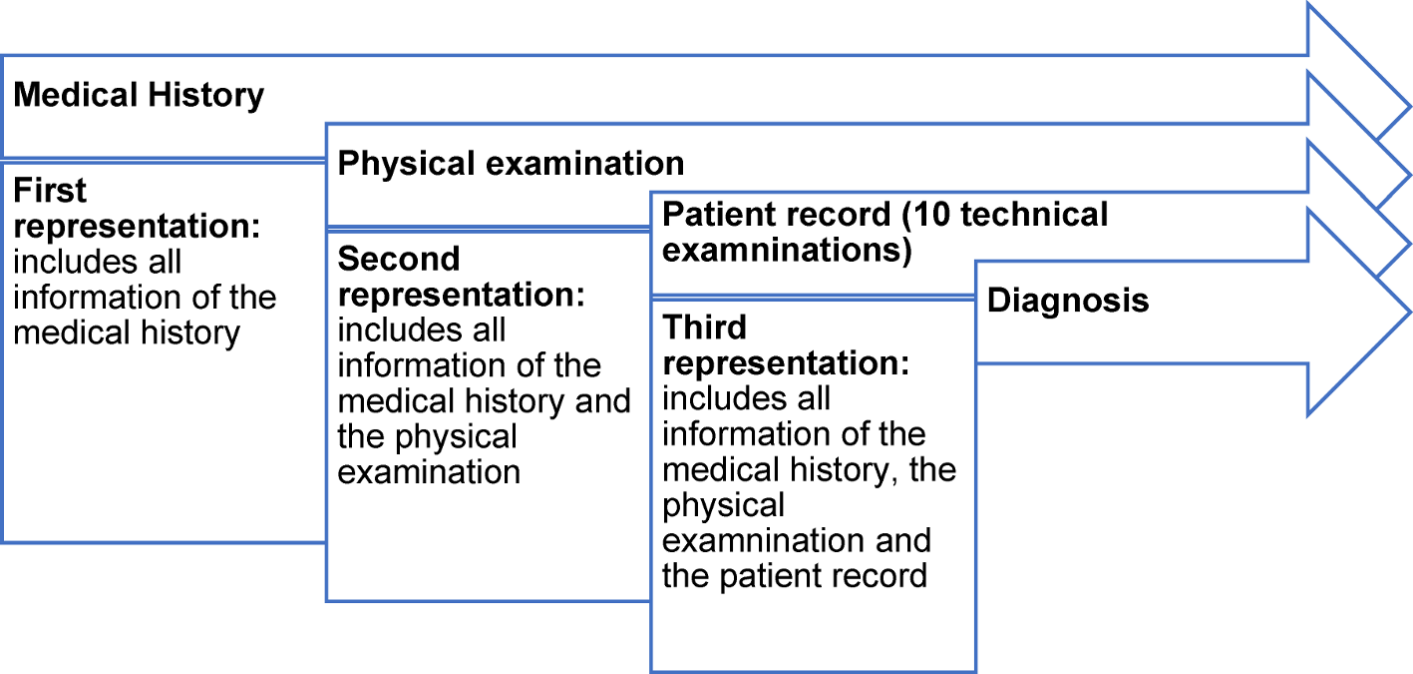
Case structure (identical in all four cases)

In June 2016, 50 fourth- and fifth-year medical students worked on four clinical cases with the leading symptom dyspnea [17] on the electronic learning platform CASUS [19], [20]. First, participants watched an introductory video explaining the correct editing process: they were asked to put themselves into a ward round situation where they had to introduce a patient to other physicians. In addition, the technical aspects of the learning platform, how the clinical information could be selected, were explained. During the study phase, participants worked on four clinical cases in a given order and were asked to write case presentations during casework. Three times – after the medical history, the physical examination, and the patient record – the students received the following prompt: “Please sum up the case as you would present it to your attending physician.” (see Figure 1 (Fig. 1)). Participants were able to navigate freely through the technical examinations and findings. They were able to collect as much clinical information as they wanted from the patient record, which contained 10 different types of technical examinations for each case, in a freely selectable order. Only the findings of the technical examinations were given (e.g., results of blood gas analysis or the values of a pulmonary function test) without an assessment. Thus, for example, the X-ray images did not include any written report, but the assessment in all examinations had to be made by the students themselves. After processing each case, participants had to write down their suspected diagnosis. 

#### Difficulty of the cases

The difficulty of the cases was tested in a pilot study with ten medical students of different levels of expertise. The four cases had a different degree of difficulty: the case aortic stenosis (#1) was simple and most students were able to solve it. The cases hyperventilation (#2) and uremia (#3) were of moderate difficulty; about half of the students were able to solve the cases correctly. The case AV-node-reentry-tachycardia (AVNRT, #4) was difficult due to this rather rare disease and the more complex electrocardiogram. Also, in the actual study, diagnostic accuracy was clearly different with regard to the different cases: the first case, aortic stenosis, was correctly solved by 80% of the participants; the second case, uremia, was solved by 60%, the third case, hyperventilation, by 56%, and the last case, AVNRT, by 5%. The diagnosis was binary coded (correct=successful and false=unsuccessful) relative to an expert solution of the case. In addition, experts agreed in advance which information was necessary in which case for the correct solution. Diagnostic result and process were evaluated with regard to the expert solution. The expert consensus is based on the assessments and discussions of the case developer (LB) as well as the reviewers of the cases (two advanced internal medicine assistants, a specialist in internal medicine and an attending physician) based on daily clinical practice.

#### Qualitative analysis and statistics

**Figure 2:**
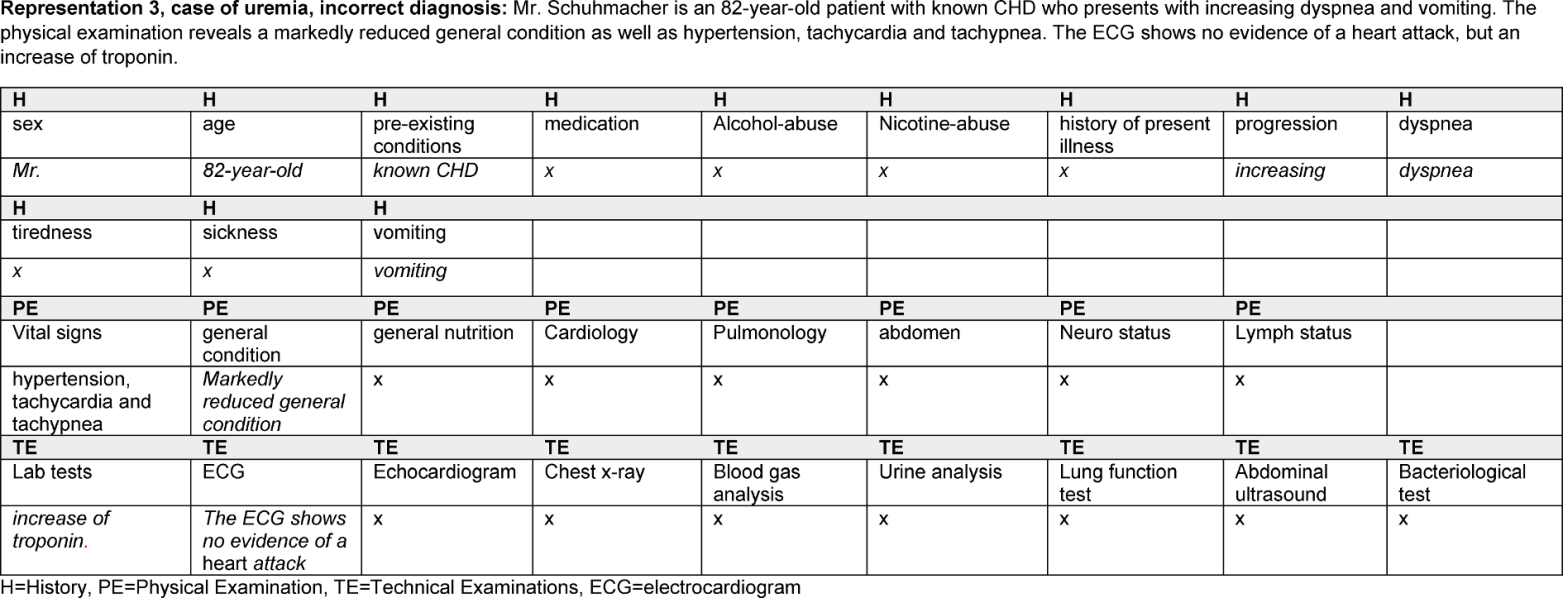
Example of a representation with incorrect diagnosis and the associated coding

**Figure 3:**
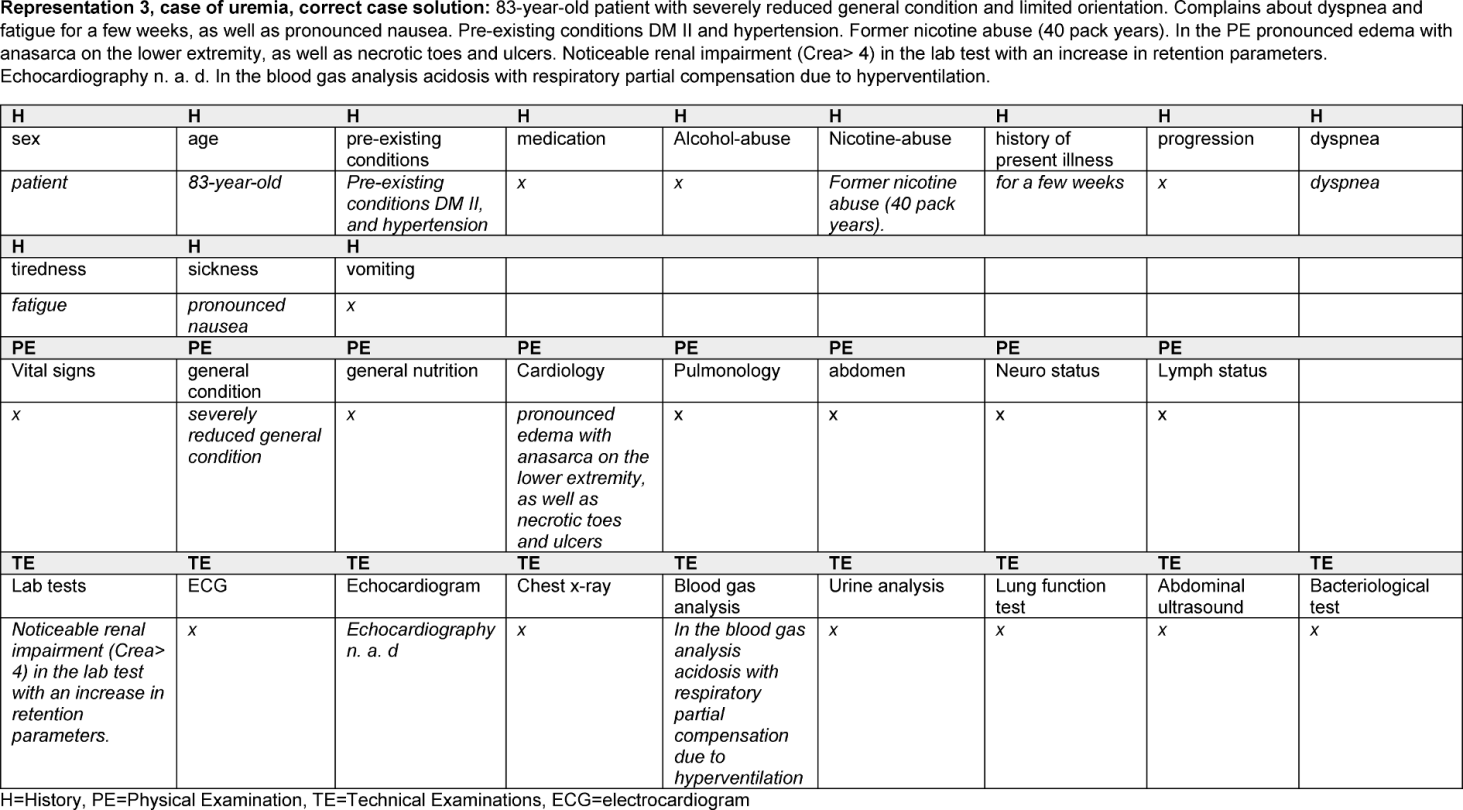
Figure 2: Example of a representation with correct diagnosis and the associated coding

In order to distinguish the different information categories in the representations, we analyzed the data based on Mayring [21]. The students’ representations were coded by assigning the contents to different categories (information levels) found in the literature [22], [23]. All representations were coded by one researcher (LB). A second rater (BL) encoded 10% of the data. The interrater coefficient (Cohen's kappa) was k=0.784. The following categories covered all information provided by the participants: **history** (including gender, age, pre-existing conditions, medication, alcohol and nicotine abuse, history of present illness, progression, symptoms), **physical examination** (vital signs, general and nutritional status, cardiovascular system, pulmonary , abdomen, neurostatus, lymph node status) and **technical examinations** (lab tests, electrocardiogram, chest x-ray, arterial blood gas analysis, lung function test, echocardiogram, urinalysis, abdominal ultrasound, bacteriological testing). It was also coded whether the participants named a diagnosis in their representation and whether they evaluated the information in any way. Two coding-examples are shown in Figure 2 (Fig. 2) and Figure 3 (Fig. 3). 

Correlations between sociodemographic data, the diagnosis result and the representations were calculated using Pearson's correlation coefficient. Group differences were tested by t-tests, Mann-Whitney tests or the Wilcoxon test. P-values≤.05 were considered statistically significant. Due to the exploratory nature of the study, we did not consider multiple testing [24].

## Results

43 students (29 women, 14 men) completed all cases; the other 7 records were incomplete and were excluded from the analysis. 516 case representations were recorded. All representations were coded with the above-mentioned coding scheme. Examples of representations of a diagnostic process that led to the correct result and one that led to an incorrect diagnosis are shown in Figures 2 (Fig. 2) and Figure 3 (Fig. 3). For all results, there were no differences between the sexes or the years of training.

### Information levels, length and dynamics of representations

Subjects could have reacted to 12 different levels of information in the first representation, 20 in the second and 30 in the third (that is, a total of 62 information levels in each of the four cases). Table 1 (Tab. 1) lists the absolute and relative amounts of information students included in their representations. The length of the representations (number of words) increases from the first to the third representation in all four cases. The first was on average M=29 words long (SD=9), the second M=34 (SD=16), while the last representation comprised M=55 words (SD=27). Although students continually received more information during case processing, they did not include more information in relation to the amount (see Table 1 (Tab. 1)). Overall, the students enriched their second representation with new clinical information, but especially in the third representation, the students selected the information and included, for example, less physiological findings in the second and third representations. The case aortic stenosis (No. 1, simple) differs slightly: already in the second representation much less clinical information is included.

The students included many anamnestic aspects (see Table 2 (Tab. 2)) in their first representation; however, the amount of anamnestic data decreased in the second and third representations. The temporal aspects are not always mentioned; especially the time line is often omitted. Also, the clinical symptoms are often incomplete. In each case, the patient presented with four different symptoms, but the students tended to name only two or fewer symptoms. For example, in the case uremia the symptoms “vomiting” and “nausea” are often missing in the representations. The findings of the physical examination are presented quite extensively in the second representation; however, they are barely mentioned in the last representation (see Table 2 (Tab. 2)). Of all 10 technical examinations, on average only the results of two are reported (see Table 2 (Tab. 2)). 

In advance, it was determined in the expert consensus, which information was absolutely necessary for the case resolution. In any case, for example only the results of a single technical investigation would have been really important to solve the case correctly. The third representation would have had to contain only 4-5 items of positive information per case. By contrast, students included significantly more items in their representations (see Table 1 (Tab. 1)). For representation 1 and 2, it was not possible to determine in advance how much information must be contained to reflect a good representation. Especially with regard to the mention of negative information or physiological results, it is unclear whether or not they should be part of the representations at the beginning of the diagnostic process.

In addition, all representations were analyzed whether a diagnosis was made or if the information was evaluated. 51% of the students never mentioned a diagnosis in any of the 12 representations. 30% named a diagnosis once, 14% twice and only 5% gave a diagnosis three times. If the students gave a diagnosis, this was most likely in the case aortic stenosis (#1).

The difficulty of the case affects the representations only slightly. The amount or type of clinical information is not case-dependent, but very similar in all four cases. Thus, the scope of a single representation seems to be a stable personality trait: the length of the representations of a single participant correlates between cases (r=0.60-0.86).

#### The representations relating to the diagnostic result

In addition, we compared the representations of the successful and unsuccessful diagnostic processes in the cases of moderate difficulty (uremia and hyperventilation). The representations of the other two cases were not compared because these cases were solved by almost all students (aortic stenosis) or very few (AVNRT). The length (number of words) does not correlate with the correct case solution (r=-0.08-0.31). In both cases, there are no differences in the first and second representations regarding the amount or type of clinical information included in the representations.

By contrast, the third representation differs between the students with a successful and those with an unsuccessful diagnostic process: The successful students included more correct information in their representations, such as the results of blood gas analysis in the case of hyperventilation (71% vs. 26%, p=0.004) or information from the history in case of uremia (time line 73% vs. 35%, p=0.015 and vomiting 73% vs. 35%, p=0.015). Furthermore, in successfully solved cases, the representations contained less unimportant or distracting information, such as the medication in the case of hyperventilation (46% vs. 84%, p=0.011) or the results of the lung function test (8% vs. 37%, p=0.024). 

## Discussion

### Purpose and summary of the study

The aim of this study was to analyze the information levels of students’ case representations. First, we tried to answer how much and what information is included in repeated representations and how this changes during the diagnostic process. Second, the relationship between the information included in the representations and the correct case solution was studied. This knowledge can contribute to a more targeted promotion of the clinical reasoning process of students.

#### The representations against the theoretical background

In this study, the strengths and weaknesses of student representation were revealed. Representations change during the diagnostic process. At first, almost all the information is included, but as the information increases, the participants begin to select and rank the clinical information. In particular, negative results of technical investigations or epidemiological aspects (e.g., alcohol consumption) that turn out to be irrelevant are no longer included in the second and third representations. This fits in with the theoretical concepts of Charlin et al. describing the dynamics of representations [6]. Nonetheless, students include far more information in their representations than would be necessary. On the one hand, the mention of physiological findings can reflect a poor diagnostic process, since the selection of the relevant information is not successful. On the other hand, physiological findings could also be deliberately cited by students (such as oxygen saturation in the case hyperventilation as opposed to the differential diagnosis of asthma attack) to delineate differential diagnoses. Based on the length of the representations, no conclusions can be drawn about the value of the representation.

First and foremost students report the clinical information, but hardly evaluate it and seldom name a diagnosis. This is consistent with other studies [5]. In addition, they have difficulty connecting the symptoms of a patient. Often only one or two symptoms are mentioned, although all four belong together (for example, in the case of uremia, the symptoms “vomiting”, “nausea”, “dyspnea“ and “tiredness”). Perhaps students did not know - or did not notice - that these symptoms combined could indicate renal insufficiency. Obviously, some students have difficulty understanding the pathophysiology of a case. In addition, the temporal course of the disease was often absent, although of course this is of great importance. This is one of the reasons why students made a wrong diagnosis: they mentioned for example suspected diagnoses, which have an acute onset, although in the history a chronic course was described.

Interestingly, the representations did not differ significantly between the individual cases, although they differed in the degree of difficulty. However, this result is surprising only at first glance: with increasing clinical experience (e.g., through the number of medical clerkships), students appear to learn e.g. epidemiological information is an obligatory part of case summaries and therefore they are listed in all representations. Regarding Bordage's description of semantic qualifiers as “the appropriate medical jargon” [13], the informational levels of student representations – regardless of the case-resolution accuracy – are very similar: students know what they should include in a representation – they follow that “appropriate medical jargon”. This could be a reason for the small differences between the individual representations.

Furthermore, there seems to be an informational maximum that can be contained in a representation. In all four cases, the students mentioned in their last representation 10-12 information levels – in the second representation, the differences between the cases were significantly larger. For a successful case solution, the case-specific medical context seems to be more important than the general representation process, which is actually mastered pretty well by the students.

#### Strengths and weaknesses

This study has several strengths. We examined a relatively large sample in a laboratory setting with very realistic cases. In addition, to our knowledge, this study is the first to analyze medical information from students’ case representations. Nevertheless, the study has limitations. We have only studied four internist cases with the main symptom of dyspnea and therefore, we cannot make any statements about case representations in other disciplines. In addition, students had to justify their diagnoses at the end of each case. This could have influenced the diagnostic process to an extent that we cannot estimate.

## Conclusions and implications for teaching

Student representations are satisfactory and dynamic at the information level. Thereby, they fulfill the required criteria for representations in the diagnostic process. Generally speaking, a representational prompt leads to fairly similar and stable representations and is independent of the difficulty of the case. From this it can be concluded that representation tasks can be mastered well by students. Scaffolds, how a representation is to be done, are not necessary for advanced medical students. Student deficits in diagnosis are more likely due to knowledge gaps. In particular, pathophysiological knowledge seems to be insufficient. Many students fail to connect symptoms or to bring in line different findings. Their knowledge is either not well-structured or retrieving the relevant facts in order to solve the case correctly, does not succeed - or both. Therefore, instructional prompts for explaining the combinations of various symptoms could be a valuable step in improving diagnostic competence.

## Competing interests

The authors declare that they have no competing interests. 

## Figures and Tables

**Table 1:**
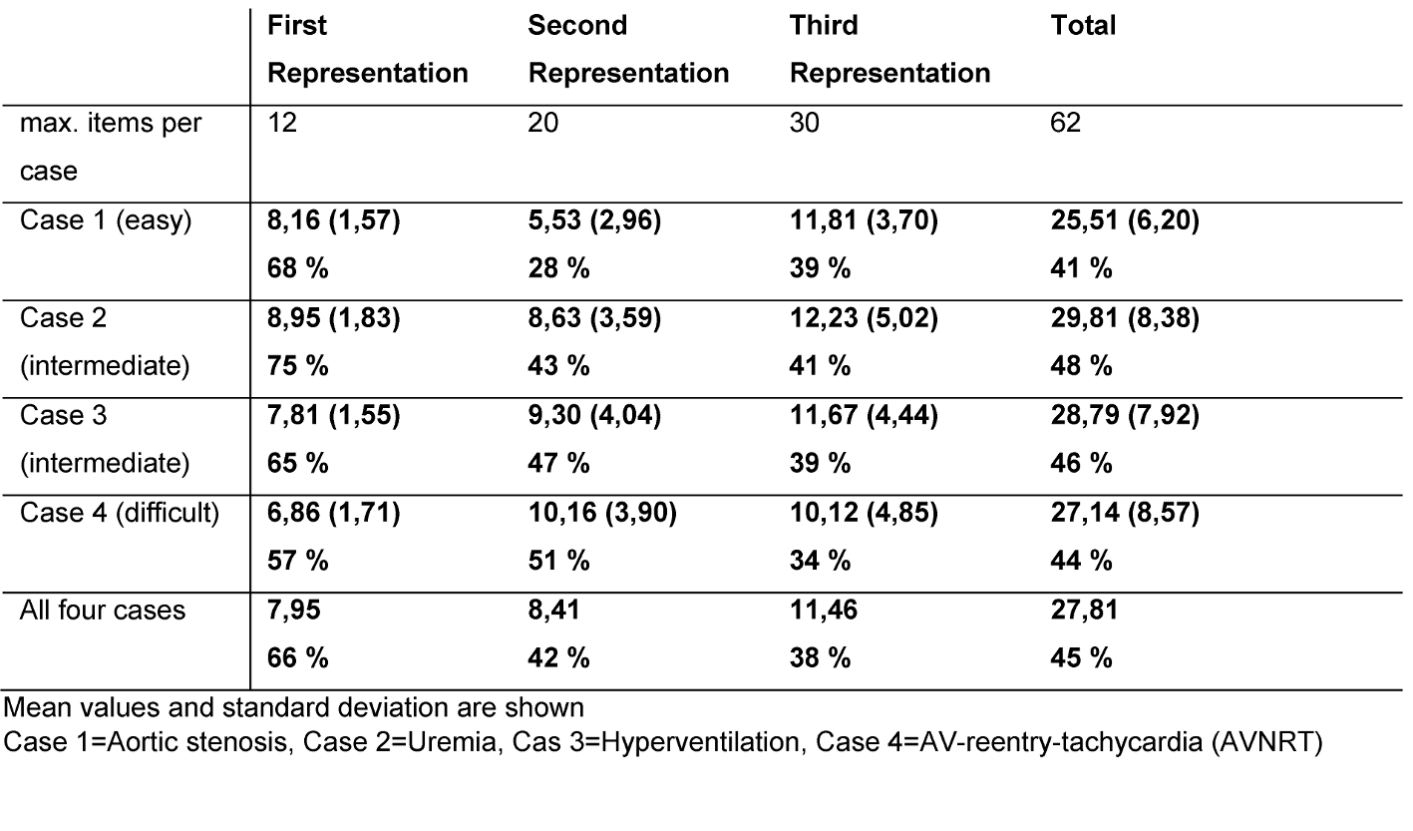
Information levels of the three representations in the four cases

**Table 2:**
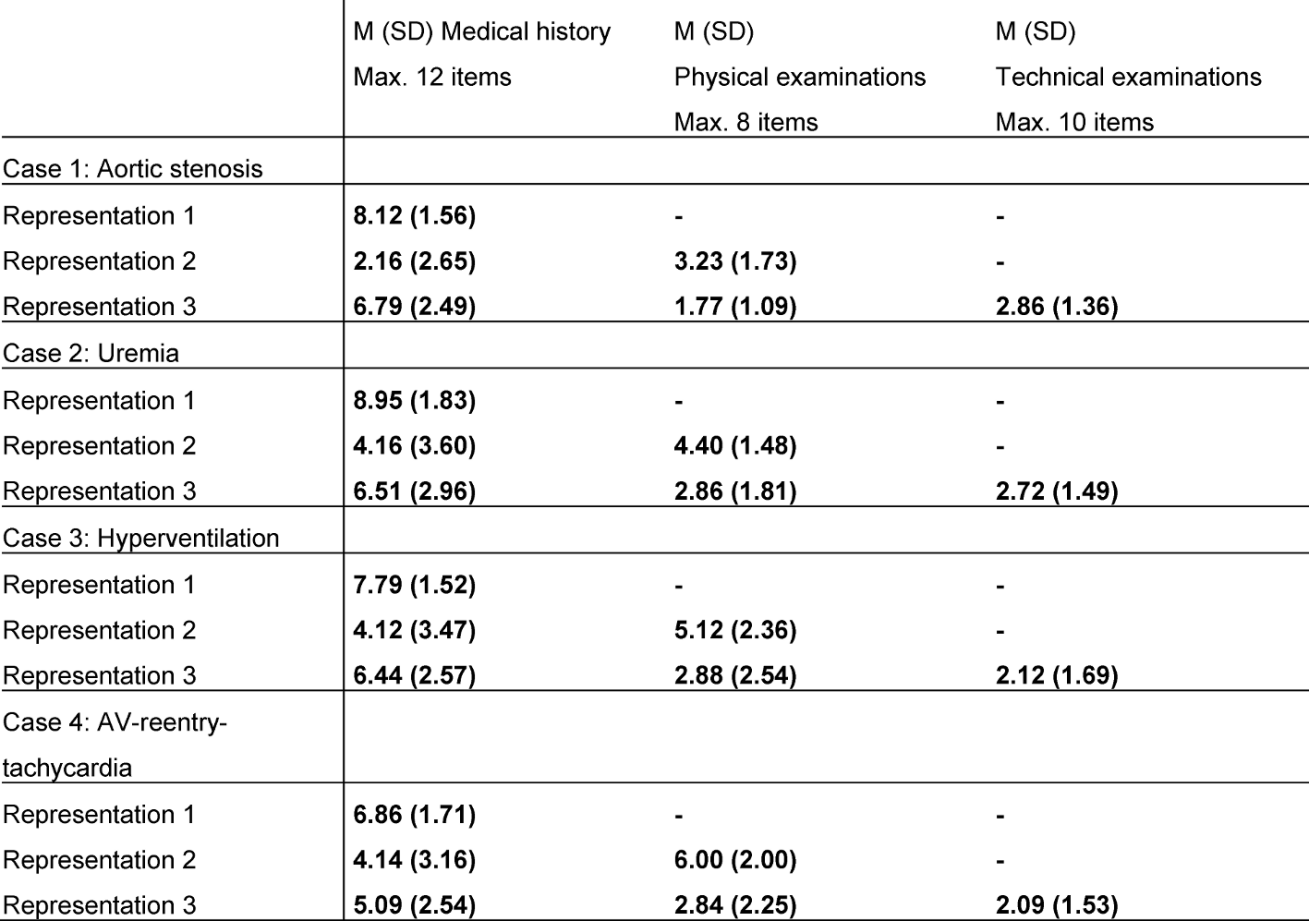
Development of the information levels
